# Cortical hyperexcitability evolves with disease progression in ALS

**DOI:** 10.1002/acn3.51039

**Published:** 2020-04-18

**Authors:** Parvathi Menon, Mana Higashihara, Mehdi van den Bos, Nimeshan Geevasinga, Matthew C. Kiernan, Steve Vucic

**Affiliations:** ^1^ Westmead Clinical School University of Sydney Sydney NSW Australia; ^2^ Brain and Mind Center University of Sydney Sydney NSW Australia

## Abstract

**Objective:**

Cortical hyperexcitability has been established as an early feature of amyotrophic lateral sclerosis (ALS). The evolution of cortical hyperexcitability with ALS progression remains to be fully elucidated. This study aims to investigate changes in cortical function in ALS with disease progression.

**Methods:**

Cortical function assessed by threshold tracking transcranial magnetic stimulation (TMS) along with clinical phenotyping was prospectively undertaken on 444 patients presenting with suspected ALS (345 ALS; 99 neuromuscular mimics). Disease stage was defined as follows: (1) King’s clinical staging system and (2) proportion of disease duration statistically categorized into tertials.

**Results:**

Cortical hyperexcitability was evident across all ALS stages, being more prominent in later stages of ALS as indicated by increased motor‐evoked potential amplitude (*P* < 0.05), as well as longer disease duration as reflected by reduced short‐interval intracortical inhibition (*P* < 0.05). Prolonged central motor conduction time was evident with disease progression. These changes were accompanied by reduction in neurophysiological index (*P* < 0.001) and compound muscle action potential amplitude (*P* < 0.01), progressive muscle weakness (*P* < 0.001), and decline in the ALS functional rating scale (*P* < 0.001).

**Interpretation:**

This study established an increase in cortical hyperexcitability with increased disease duration in ALS, mediated by cortical disinhibition and direct increase in corticomotoneuronal excitability.

## Introduction

Amyotrophic lateral sclerosis (ALS) is an adult‐onset progressive neurodegenerative disorder of the upper and lower motor neurons.^1^ The pathophysiological processes underlying ALS development are complex and heterogenous, although the extent to which each neuronal populations are affected varies across individuals and disease sages.^2^ Cortical hyperexcitability appears to be an important pathophysiological process in ALS, contributing to neurodegeneration via an anterograde glutamatergic excitotoxic process.^1^, ^3^ Support for this hypothesis is provided by transcranial magnetic stimulation (TMS) studies disclosing that cortical hyperexcitability is a specific and early feature of ALS, which precedes onset of muscle weakness, underlies development of clinical signs such as the split hand phenomenon, contributes to disease progression, and correlates with greater functional disability.^4^, ^5^, ^6^, ^7^, ^8^ Importantly, there appears to be a variability in the degree of cortical hyperexcitability with disease evolution in ALS,^9^ suggesting a vulnerability of specific cortical interneurons across different disease stages.

Cortical hyperexcitability is heralded by reduction or absence of short‐interval intracortical inhibition (SICI), leading to disinhibition and hyperexcitability of the cortical motor neurons,^10^ despite a recent study suggesting that spinal motor neuron excitability was normal or reduced in ALS.^11^ Given that SICI is mediated by cortical inhibitory interneurons acting via GABA_A_ receptors,^12^ it has been surmised that dysfunction of these cortical inhibitory interneurons contributes to SICI abnormalities and thereby development of cortical hyperexcitability in ALS.^13^ At a pathological level, degeneration of parvalbumin and calbindin D‐28k‐positive inhibitory cortical interneurons^14^ along with reduced expression of GABA_A_ alpha 1 subunits,^15^, ^16^ has been documented in ALS patients, all contributing to reduction in SICI and thereby cortical hyperexcitability.

The relationship between cortical hyperexcitability with disease stage, as defined by King’s criteria and proportion of disease duration, remains to be fully defined. Transcranial magnetic stimulation studies conducted on smaller ALS cohorts reported a loss of inhibitory circuits with disease progression, although phenotypic categorization was limited in these studies.^17^, ^18^, ^19^ More recently, a decline in SICI was reported with disease progression in a large and well‐phenotyped ALS cohort,^9^ although the relationship of cortical dysfunction with the King’s staging system was not investigated. Consequently, this study investigated whether cortical dysfunction changes across different stages of ALS, as defined by the King’s staging system, and whether longitudinal changes in cortical function develops in ALS.

## Methods

### Subjects

Patients were prospectively recruited from two neuromuscular centers, integrated through Sydney Health Partners University of Sydney, following detailed assessment. The patients were consecutively recruited in accordance with the inclusion criteria, which included: (i) pure lower motor neuron syndrome; (ii) mixed upper and lower motor neuron syndrome; or (iii) upper motor neuron syndrome. Written informed consent for the procedures was provided by all subjects and approved by both the Western Sydney Local Health District.

### Clinical assessment

The diagnosis of sporadic ALS was made in accordance to the Awaji criteria.^20^ Familial ALS patients were excluded from the study. All patients underwent detailed clinical assessment at recruitment including recording the site of disease onset, disease duration from symptom onset (months), muscle strength (Medical Research Council [MRC]^21^ score), and upper motor neuron (UMN) score.^22^ The following muscles were assessed bilaterally: shoulder abduction, elbow flexion/extension, wrist dorsiflexion, finger and thumb abduction, hip flexion, knee extension, and ankle dorsiflexion, yielding a maximum MRC score of 90 (indicating normal strength). Disease severity was assessed by using the ALS functional rating scale (ALSFRS‐R)^23^ and the rate of disease progression.^24^

ALS patients were staged according to a modified King’s staging system,^25^ which included: ***stage 1***, involvement of one clinical region; ***stage 2***, involvement of two clinical regions; ***stage 3***, involvement of three clinical regions; ***stage 4***, nutritional or respiratory failure; and ***stage 5***, death. The previously defined stage 2A (diagnosis) and stage 2B (involvement of second region) were combined into stage 2, in order to facilitate statistical analysis.

### Neurophysiological studies

Prior to undertaking cortical excitability studies, the median nerve was electrically stimulated at the wrist. The ensuing compound muscle action potential (CMAP) was recorded from the abductor pollicis brevis (APB) using AgCl gel disc electrodes (10 mm diameter 3M). The ground electrode placed over the dorsum of the same hand. The peak–peak CMAP amplitude, distal motor latency, and F‐wave frequency were recorded, and neurophysiological index (NI) was calculated from the median nerve in accordance with a previously reported formula.^26^ Studies were undertaken on the dominant motor cortex in both ALS and non‐ALS pathological controls.

Cortical function was assessed in all patients by the threshold tracking TMS technique according to previously reported methodology.^27^ A 90‐mm circular coil was used to stimulate the primary motor (M1) cortex, with coil position adjusted to generate an optimal motor evoked potential (MEP). The minimum median nerve CMAP amplitude for required for TMS to proceed was >1 mV. The following TMS parameters were recorded: (i) resting motor threshold (%); (ii) short‐interval intracortical inhibition (SICI, %), over interstimulus intervals (ISIs) of 1, 1.5, 2, 2.5, 3, 3.5, 4, 5, and 7 msec; (iii) intracortical facilitation (ICF), over ISIs 10, 15, 20, 25, and 30 msec; (iv) maximal MEP amplitude (% CMAP amplitude); and (v) cortical silent period (CSP) duration.^28^ The TMS intensity was set to 150% RMT for determination of MEP amplitude and CSP duration, and three responses were recorded at this stimulus intensity.^27^

### Statistical analyses

Clinical and neurophysiological findings in ALS patients were compared to 99 non‐ALS controls. The differences in clinical and neurophysiological parameters were assessed across the King’s clinical staging system.^25^ In addition, the neurophysiological parameters were also related to proportion of disease duration, defined as statistically derived tertials as follows: *short duration* <8 months; *intermediate* 8–17 months; and *longer duration* >17 months. Student’s *t*‐test was used to assess differences between means. One‐way analysis of variance or Kruskal–Wallis test was used for multiple comparisons. Pearson correlation was used to assess whether there were any associations between neurophysiological and clinical parameters. All data are expressed as mean ± standard error of the mean or median (interquartile range). *P* value <0.05 was deemed significant.

## Results

### Clinical features

Between 01 January 2010 and 01 September 2019, 444 patients (268 males, 176 females, mean age 59.2 ± 0.6 years) satisfying the inclusion criteria were prospectively recruited. In total, 345 patients were diagnosed with ALS, with 27.2% patients classified as Awaji definite, 29% as probable, 29% possible, and 14.8% did not meet the Awaji criteria. All Awaji negative patients progressed to the Awaji definite or probable diagnostic categories at the census date (01 September 2019). Twenty‐six (6%) patients could not undergo TMS testing due to marked wasting of the recording muscle. Separately, 99 patients were diagnosed with a non‐ALS neuromuscular mimic disorder and served as pathological controls.

Mean disease duration in ALS patients at time of assessment was 17.5 ± 1.0 months, with 68% of patients exhibiting limb‐onset and 32% bulbar‐onset disease. At time of TMS testing 40% of ALS patients were classified as King’s stage 1, 29% as stage 2, 30% as stage 3, and 1% as stage 4. The disease duration was similar across the King’s clinical stages (*F* = 1.05, *P* = 0.37, Table 1). Riluzole therapy was administered to 48% of patients at time of TMS testing, with 33% being classified as King’s stage 1, 23% stage 2, and 43% as stage 3. In addition, ALS patients were older (*P* < 0.001), exhibited more prominent UMN signs (*Z* = −9.08, *P* < 0.001), and exhibited a greater degree of muscle weakness (*Z* = −6.417, *P* < 0.001) when compared with controls [Table 1]. In addition, the UMN score was significantly higher in King’s stages 2 and 3 when compared with stage 1 patients (*χ* ^2^ = 21.518, *P* < 0.001, Table 1), while the total MRC score was significantly lower in stages 2 and 3 patients (*χ* ^2^ = 32.928, *P* < 0.001, Table 1).

**Table 1:** Clinical findings in ALS.

	ALS Cohort	King’s stage 1	King’s stage 2	King’s stage 3	Non‐ALS
Mean age at assessment [years] (SEM)	61.4 (0.6)	61.3 (1.0)	61.0 (1.1)	61.8 (1.1)	51.6 (1.6)
Mean disease duration [months] (SEM)	17.5 (1.0)	18.6 (1.7)	15.0 (1.8)	18.6 (1.7)	91.9 (12.9)
Mean ALSFRS‐R	40.5 (0.3)	41.8 (0.6)	40.2 (0.4)	38.8 (0.6)	Not done
Mean rate of disease progression	0.8 (0.04)	0.58 (0.1)	0.88 (0.1)	0.84 (0.1)	Not done
Median UMN score	11 (4–13)	8 (0–12)	11 (6–13)	12 (9–13)	0
Median MRC sum score	82 (76–88)	86 (80–90)	81 (74–85)	80 (71.5–86)	89 (86–90)

Clinical features of amyotrophic lateral sclerosis (ALS) patients and non‐ALS pathological controls. The neuromuscular mimic disorder group included acquired neuromyotonia syndrome (23), hereditary spastic paraplegia (12), myopathy (12), Kennedy’s disease (9), Hirayama’s disease (8), pure motor chronic inflammatory demyelinating neuropathy (7), multifocal motor neuropathy (7), spinal muscular atrophy (6), FOSMN syndrome (4), distal hereditary motor neuronopathy with pyramidal features (3), post‐polio syndrome (2), cervical radiculopathy (2), Pompe disease (2), lumbosacral radiculopathy (1), and lead toxicity (1). All patients were assessed using the Medical Research Council (MRC) score and upper motor neuron (UMN) score. All data are expressed as mean (standard error of the mean) or median (interquartile range).

The mean ALSFRS‐R score in the ALS cohort was 40.5 ± 0.3, indicating mild–moderate degree of functional disability. As expected, the ALSFRS‐R score was significantly lower in stage 3 ALS patients when compared with stages 1 and 2 (*F* = 3.352, *P* < 0.01, Table 1). In addition, the rate of disease progression was significantly higher in King’s stages 2 and 3 ALS patients when compared with stage 1 patients (*F* = 3.668, *P* < 0.05, Table 1).

### Neurophysiological studies

#### Lower motor neuron (LMN) function

Prior to undertaking assessment of cortical excitability, the degree of LMN dysfunction was determined. The CMAP amplitude was significantly reduced in ALS patients (6.8 ± 0.2 mV) when compared with pathological controls (8.6 ± 0.4 mV, *F* = 13.3, *P* < 0.001). Subgroup analysis disclosed that the reduction in CMAP amplitude was only significant in stage 2 (*P* < 0.001) and stage 3 (*P* < 0.001, Table 2) ALS patients when compared with controls, with King’s stage 1 patients exhibiting larger CMAP amplitudes when compared with stage 2 (*P* < 0.05) and stage 3 (*P* < 0.01) patients.

**Table 2:** Summary of neurophysiological findings.

	ALS Cohort	King’s stage 1	King’s stage 2	King’s stage 3	Non‐ALS
Mean CMAP amplitude (mV)	6.8 (0.2)	7.7 (0.4)	6.4 (0.3)	6.1 (0.4)	8.6 (0.4)
Mean NI	1.3 (0.1)	1.7 (0.1)	1.2 (0.1)	0.8 (0.1)	2.1 (0.3)
Mean averaged SICI (%)	3.0 (0.5)	2.4 (0.9)	3.8 (1.1)	3.3 (0.9)	11.9 (0.6)
Mean ICF (%)	−2.7 (0.4)	−3.3 (0.7)	−2.8 (0.8)	−1.9 (0.6)	1.1 (0.6)
Mean RMT (%)	57.9 (0.6)	58.7 (1.0)	58.1 (1.2)	56.7 (1.2)	56.2 (1.0)
Mean MEP amplitude (%CMAP)	38.6 (1.4)	34.8 (2.1)	34.7 (2.3)	45.5 (3.1)	27.7 (1.9)
Mean CSP duration (ms)	180.4 (2.6)	180.9 (4.5)	182.5 (4.7)	182.5 (4.8)	208 (3.8)
CMCT (ms)	6.8 (0.2)	6.2 (0.2)	6.5 (0.2)	6.3 (0.3)	6.2 (0.4)
ALSDI	12.8 (0.5)	13.3 (0.9)	12.7 (1.0)	12.2 (0.9)	4.6 (0.3)

Neurophysiological features for amyotrophic lateral sclerosis (ALS) patients and non‐ALS pathological controls. The median nerve compound muscle action potential (CMAP) amplitude, neurophysiological index (NI), short‐interval intracortical inhibition (SICI), resting motor threshold (RMT), motor‐evoked potential (MEP) amplitude, cortical silent period (CSP) duration, central motor conduction time (CMCT), and ALS diagnostic index (ALSDI) scores are depicted. All data are expressed as mean (standard error of the mean).

The NI was significantly reduced in ALS patients (1.3 ± 0.1) when compared with controls (2.1 ± 0.3, *F* = 7.953, *P* < 0.001). The reduction in NI was significant in King’s stages 2 (*P* < 0.01) and 3 (*P* < 0.001, Table 2) patients when compared with controls.

#### Cortical function

**Figure 1:**
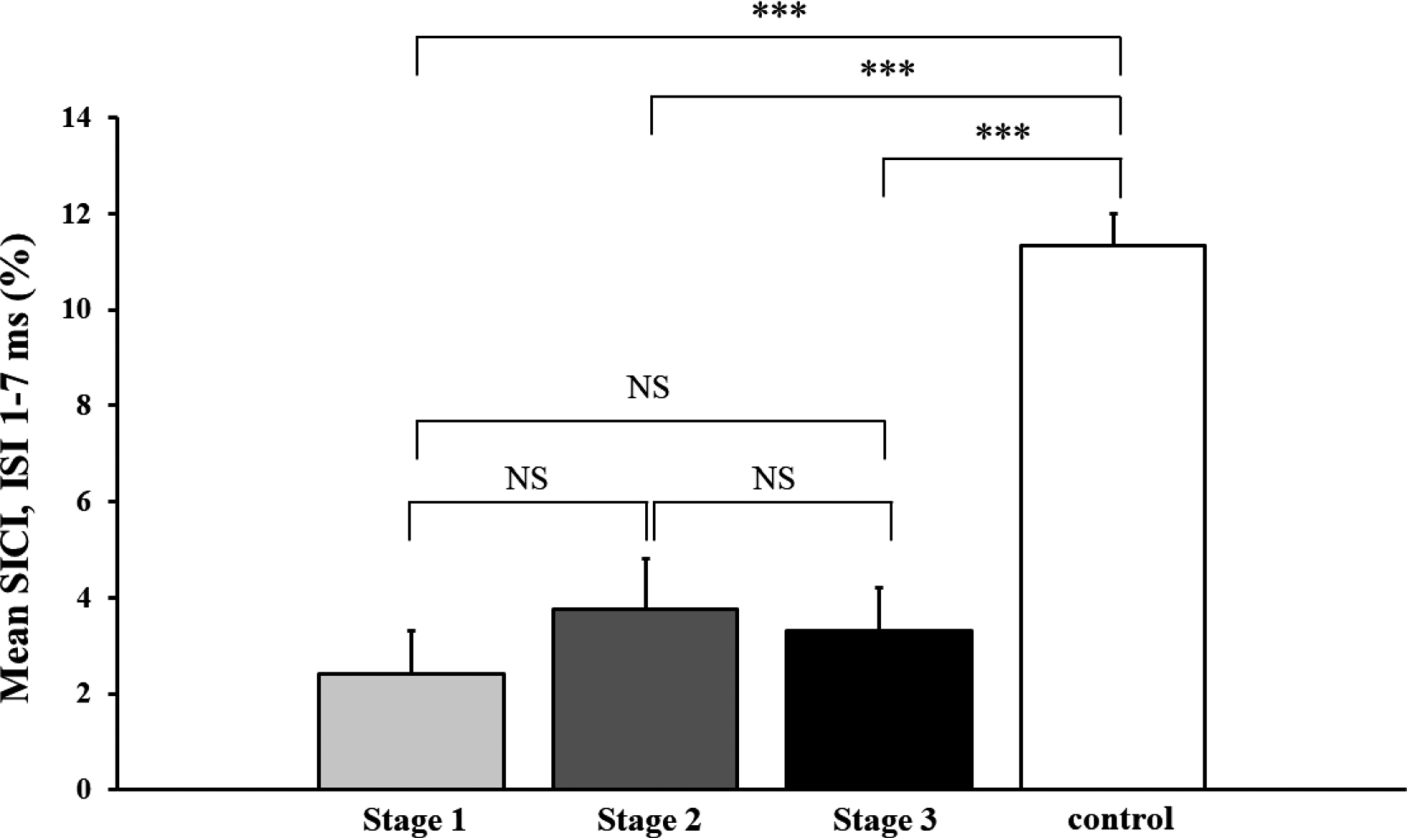
There was a significant reduction in mean short‐interval intracortical inhibition (SICI), between interstimulus intervals (ISI) 1 and 7 msec, in amyotrophic lateral sclerosis (ALS) patients when compared with neuromuscular controls. The reduction in SICI was comparable across the King’s clinical stages in ALS patients. NS, Nonsignificant; ***P < 0.001.

Inexcitability of the motor cortex, defined as stimulator output required to elicit an MEP response of 0.2 mV >90% of maximum stimulator output, was evident in 13% of ALS patients. In a further 9% of the cohort, recording of MEPs and CSPs was not possible as the RMT was >70%. The frequency of motor cortex inexcitability was similarly distributed across the three King’s stages (stage 1, 4%; stage 2, 5%; and stage 3, 4%). Consequently, TMS testing was possible in 299 (81%) ALS patients. Inexcitability of the motor cortex was not evident in neuromuscular controls and RMT was comparable to ALS patients (*P* = 0.09, Table 2). The RMT was similar across the three King’s stages (*F* = 1.939, *P* = 0.123, Table 2). There was a marked reduction in mean SICI in ALS patients (3.0 ± 0.5%) when compared with controls (11.9 ± 0.6% *P* < 0.001). The reduction in SICI in King’s stage 1 patients was comparable to that evident in stages 2 and 3 patients (Fig. 1, Table 2). The frequency of ALS patients exhibiting cortical hyperexcitability, defined as SICI < 5.5%,^10^ was similar across the three King’s stages (*P* = 0.91), as was the proportion of ALS patients with absent SICI (*P* = 0.69).

**Figure 2:**
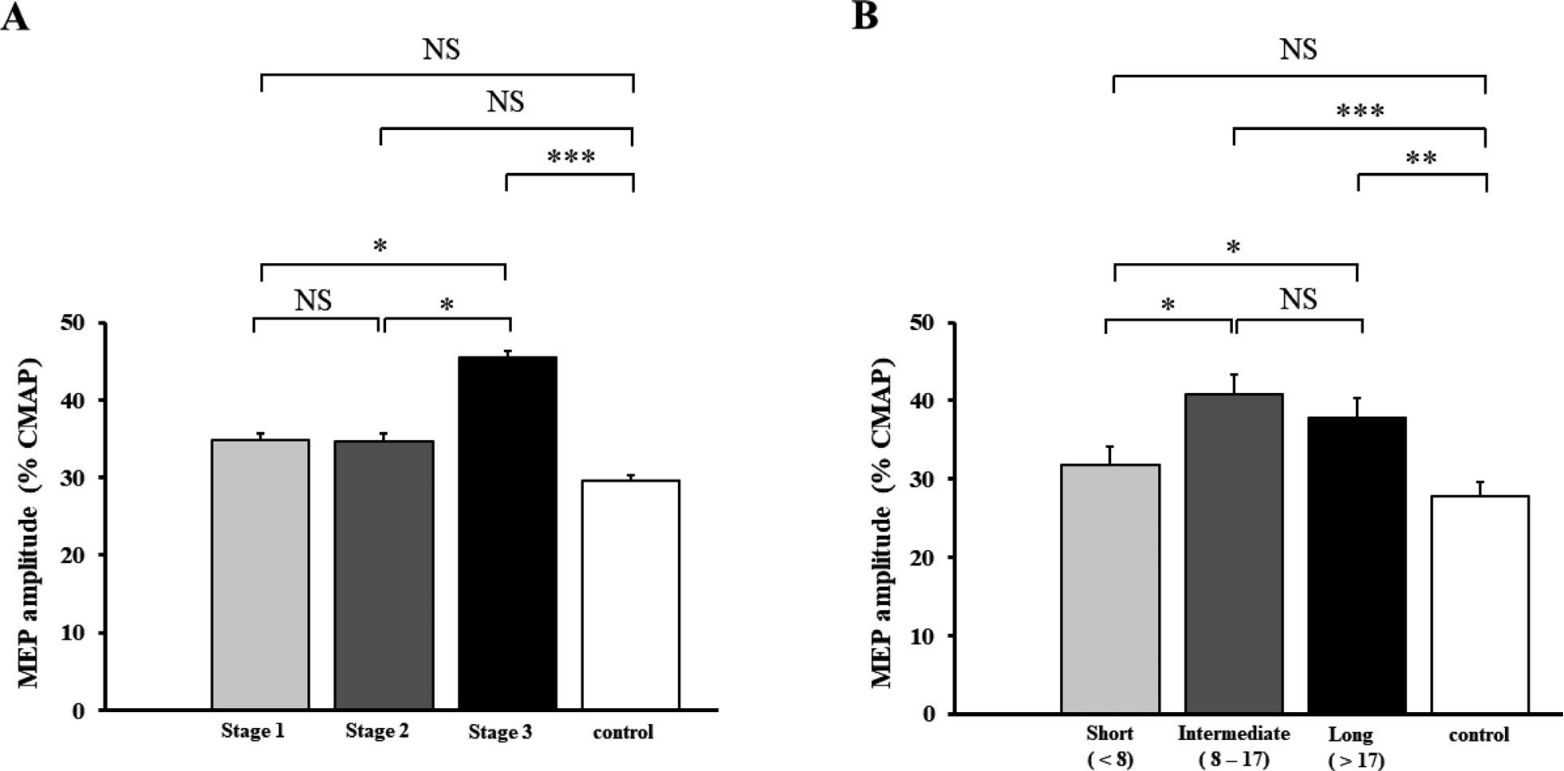
Longitudinal studies in a cohort of amyotrophic lateral sclerosis (ALS) patients (*N* = 23) disclosed a significant reduction in mean short‐interval intracortical inhibition (SICI), between interstimulus intervals (ISI) 1 and 7 msec, with greater disease duration. NS, Nonsignificant; *P < 0.05.

Given that disease duration was similar across the King’s stages, the ALS cohort was divided into three equal groups according to disease duration as outlined in the methods. The mean SICI was significantly smaller in the patients with longer disease duration (1.5 ± 0.8%, *P* < 0.05) when compared with those with short (4.2 ± 0.9%) and intermediate (3.8 ± 1.1%, *P* < 0.05) disease durations (Fig. 2).

Intracortical facilitation follows SICI, developing between ISIs of 10–30 msec. There was a significant increase in ICF in ALS patients when compared with pathological controls (*F* = 8.745, *P* < 0.001, Table 2). The increase in ICF was comparable across the three King’s staging categories (Table 2) and between the short, intermediate, and long disease duration ALS cohorts (*F* = 0.9; *P* = 0.40).

**Figure 3:**
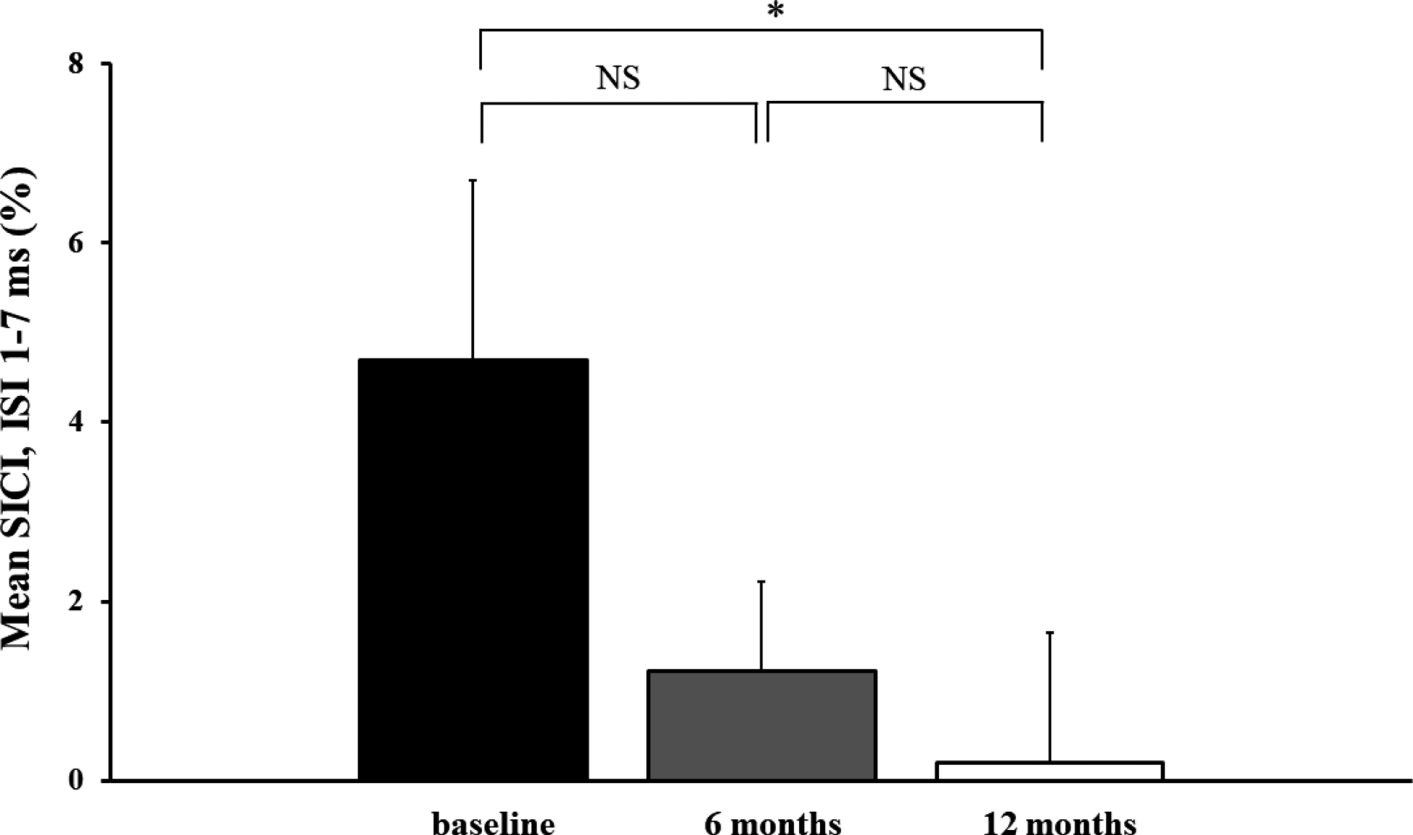
**(**A) The motor‐evoked potential (MEP) amplitude was significantly increased in amyotrophic lateral sclerosis (ALS) patients classified as King’s stage 3 when compared with King’s stages 1, 2, and neuromuscular controls. (B) The increase in MEP amplitude was significant in ALS patients classified as intermediate and longer disease duration. NS‐ Nonsignificant; *P < 0.05; ***P < 0.001.

Single‐pulse TMS revealed a significant increase in MEP amplitude, expressed as percentage of CMAP amplitude, in ALS patients (38.6 ± 1.4%) when compared with controls (27.7 ± 1.9%, *F* = 8.721, *P* < 0.001). The increase in MEP amplitude was significant across the three King’s stages (Fig. 3A, Table 2), being most pronounced in King’s stage 3 patients (Fig. 3A, Table 2). In addition, the MEP amplitude was significantly higher in ALS patients with intermediate and longer disease durations compared with those with shortest disease duration (Fig. 3B).

The CSP duration was significantly reduced in ALS patients (ALS, 180.4 ± 2.6 msec; controls 208.0 ± 3.8 msec, *F* = 7.347, *P* < 0.001). The reduction in CSP duration was similar across the three King’s stages (Table 2) and the three disease duration groups (*P* = 0.35). Separately, the central motor conduction time (CMCT) was significantly prolonged in ALS patients (6.8 ± 0.2 msec) when compared with controls (6.2 ± 0.4 msec, *P* < 0.001). Interestingly, the CMCT was only significantly increased in ALS patients with longer disease duration (6.8 ± 0.3 msec, *P* < 0.05). The changes in cortical excitability across the Kings clinical stages and disease duration stages are summarized in Figure 3.

### Correlation studies

Correlation studies were undertaken to determine any associations between clinical and neurophysiological parameters. There were no significant correlations between neurophysiological parameters (SICI, ICF, CSP duration, NI, or CMAP amplitude) and clinical measures (ASLFRS‐R, rate of disease progression, and disease duration).

### Longitudinal studies

**Figure 4:**
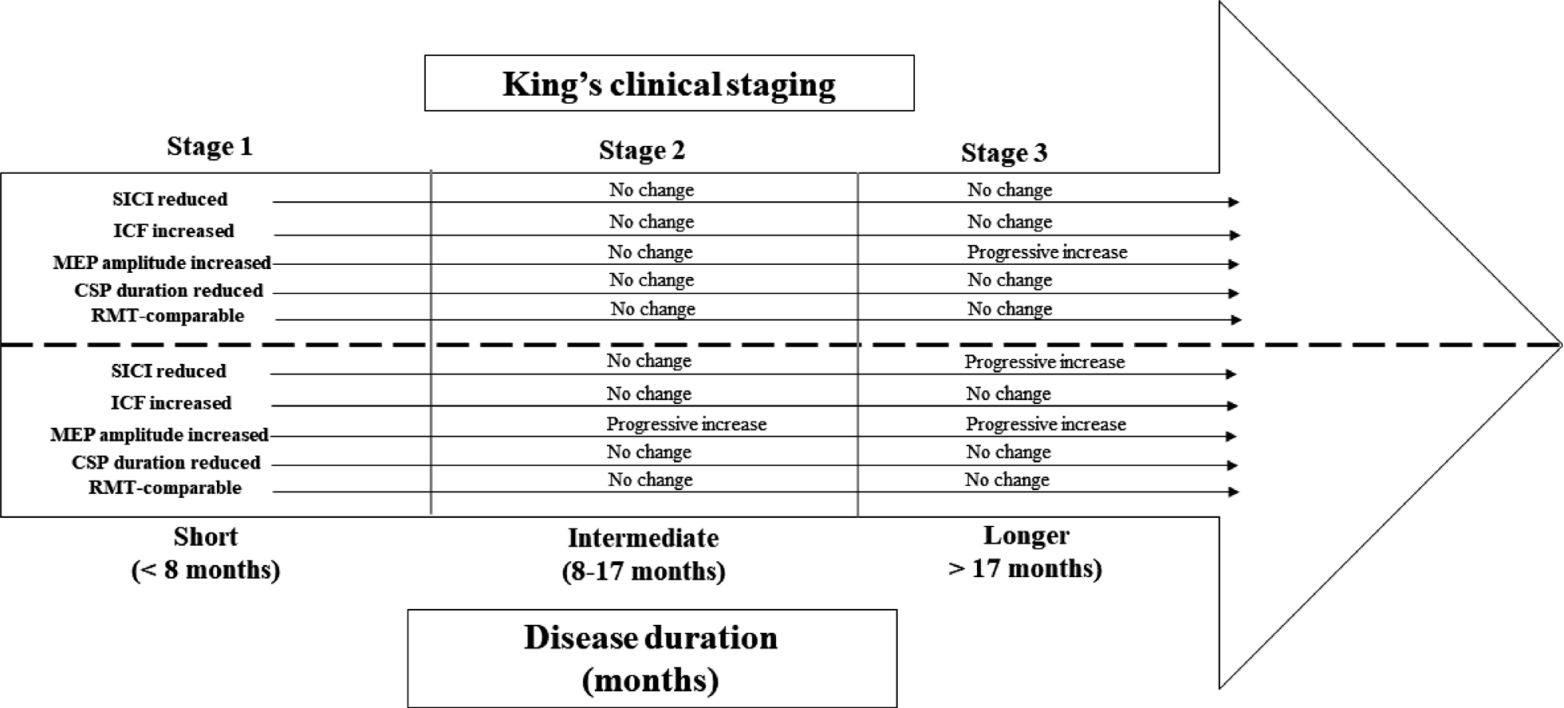
Changes in transcranial magnetic stimulation (TMS) parameters with Kings clinical stages and disease duration. Short‐interval intracortical inhibition (SICI) was significantly increased at baseline and remained stable across King’s stages but increased with disease duration. Intracortical facilitation (ICF) was increased while cortical silent period (CSP) duration was reduced at baseline and did not change with Kings clinical stages or disease duration. The motor‐evoked potential (MEP) amplitude was increased at baseline and increased with Kings clinical stages and disease duration. The resting motor threshold (RMT) was comparable with controls and did not evolve with Kings clinical stage or disease duration.

In order to further determine whether cortical excitability changes in ALS are related to disease progression, longitudinal studies were undertaken in a subgroup of ALS patients (*N* = 23; 12 males, 11 females) with cortical excitability testing performed at 6 and 12 months after baseline TMS. At baseline, mean disease duration was 24 ± 5.6 months, median MRC score was 86.5 [83–88], with 21% classified King’s stage 1, 61% stage 2, and 17% stage 3. In addition, 78% of patients were classified as Awaji possible ALS, while 32% as probable. The mean ALSFRS‐R at baseline was 42.9 ± 0.9 and mean rate of disease progression 0.42 ± 0.9. The ALSFRSR decreased to 39.8 ± 1. 5 (*P* < 0.01) over the follow‐up period. The mean SICI at baseline in the subgroup of ALS patients was 4.7 ± 2.0%, mean ICF −2.1 ± 1.4%, MEP amplitude 30.5 ± 3.4%, RMT 53.5 ± 1.7%, and CSP duration 179.2 ± 8.2 msec. There was a significant reduction in mean SICI during the follow‐up period (Fig. 4). There was no significant correlation between neurophysiological parameters and rate of disease progression at baseline.

## Discussion

This study established cortical hyperexcitability as an early feature of ALS which evolved with disease progression, as defined by the King’s clinical staging system and proportion of disease duration. Specifically, reduction in SICI and CSP duration along with an increase in MEP amplitude were indicative of cortical hyperexcitability. The increase in MEP amplitude and reduction in SICI were more prominent in later stages of ALS. Longitudinal studies disclosed a decline in SICI in a cohort of ALS patients re‐affirming the evolution of cortical hyperexcitability with disease progression. These findings suggest that cortical hyperexcitability is an early feature of ALS, and evolves disease progression, being is mediated by cortical disinhibition, and a direct increase in corticomotoneuronal excitability.

### Cortical hyperexcitability and disease progression in ALS

Cortical hyperexcitability appears to be an important pathogenic and diagnostic biomarker of ALS, mediated by an imbalance of inhibitory and facilitatory cortical circuit activity along with increased cortical neuronal excitability.^1^, ^3^, ^8^ The evolution of cortical excitability changes with disease progression in ALS remains to be fully elucidated. Previous TMS studies utilizing a cross‐sectional study design have disclosed conflicting results, with one reporting a reduction in SICI with disease progression^17^ while another study failed to establish any reductions.^18^ More recently, a reduction in SICI was reported with disease progression, as defined by proportion of disease duration and the ALSFRS‐R, in a large cohort of ALS patients.^9^ These findings suggest a progressive dysfunction of cortical GABA‐ergic inhibitory interneuronal circuits in ALS with disease evolution.^9^

The findings in this study provide additional support for evolution of cortical dysfunction in ALS. Specifically, a greater degree of SICI reduction was evident in ALS patient with longer disease duration (>17 months), underscoring the possibility that dysfunction of inhibitory interneuronal circuits, mediated by GABA_A_ receptors, are more prominent in later stages of ALS.^9^ The progressive decline in SICI on longitudinal studies provides further support for this notion. Interestingly, there was comparable reduction in SICI across the King’s clinical stages, suggesting that cortical disinhibition is an early phenomenon in ALS as previously suggested.^4^, ^5^, ^29^ Given that disease duration was comparable across the King’s clinical stages, the absence of a significant difference in SICI may be expected and does not invalidate the notion of a progressive decline in cortical inhibition in later stages of ALS. It should be acknowledged, however, that ALS patients undergoing longitudinal studies exhibited milder disease at baseline (rate of disease progression of 0.25/month), potentially confounding the results. Separately, riluzole partially and transiently normalizes SICI in ALS patients.^30^, ^31^ In this study, 48% of ALS patients were receiving riluzole at the time of TMS testing, thereby potentially impacting the results. The Kings clinical staging system was applied retrospectively, further impacting the findings.

A reduction in CSP duration was evident in the current ALS cohort, which was comparable across the King’s clinical stages and disease duration categories. The CSP duration is mediated by a combination of spinal inhibitory mechanisms and long latency inhibitory cortical processes acting via acting GABA_B_ receptors.^28^, ^32^, ^33^, ^34^, ^35^, ^36^ Consequently, dysfunction of longer latency inhibitory GABA_B_ circuits appears to be an early feature of ALS, contributing to cortical hyperexcitability, and does not evolve with disease progression.

There was a direct increase in corticomotoneuronal hyperexcitability as heralded by an increase in MEP amplitude. The MEP amplitude reflects the density and excitability of corticomotoneuronal projections onto motor neurons.^37^ Increase in MEP amplitude was evident across all King’s clinical stages, being most prominent in King’s clinical stage 3, as well as ALS patients with intermediate and longer disease duration. The findings imply that a combination of cortical disinhibition along with direct increase in corticomotoneuronal excitability underlies the development of cortical hyperexcitability in ALS. Importantly, although cortical hyperexcitability is evident in early stages of ALS, it becomes more prominent with disease progression, a finding that could be of diagnostic and therapeutic significance.

In parallel to the evolving cortical changes, there was a progressive decline in clinical and functional measures of ALS with disease progression. Specifically, a greater degree of muscle weakness and LMN dysfunction along with more prominent UMN signs was more evident in later stages of ALS. These clinical changes were accompanied by a significant reduction in the ALSFRS‐R score indicating greater functional decline. The findings that cortical hyperexcitability was evident in early stages of ALS and evolved with LMN lend credence to the hypothesis that cortical hyperexcitability is an important pathophysiological mechanism of ALS.^1^, ^38^ Although it could be argued that changes in cortical hyperexcitability were compensatory, this seems unlikely given the normal cortical excitability in non‐ALS mimics and previous studies identifying cortical hyperexcitability as a specific feature of ALS.^10^ Treatment of cortical hyperexcitability is likely to be beneficial in ALS, as indicated by continue clinical effectiveness of riluzole in later stages of ALS.^39^ A prospective longitudinal study in a larger ALS cohort may further clarify these issues.

## Author Contributions

PM contributed to conception and design of the study, acquisition and analysis of data, and drafting a significant portion of the manuscript or figures. MH contributed to conception and design of the study and acquisition of data. MvB contributed to conception and design of the study and acquisition of data. NG contributed to conception and design of the study and acquisition of data. MK contributed to conception and design of the study and editing the manuscript. SV contributed to conception and design of the study, analysis of data, and drafting a significant portion of the manuscript.

## Conflict of Interest

Prof MCK reports being editor of Journal of Neurology Neurosurgery and Psychiatry. Prof SV reports receiving honoraria from Merck Serono Australia, CSL Australia, and Biogen Pty Ltd Australia. There are no other conflicts of interest to report.
